# The 2-Step Mendelian Randomisation Study Assesses Genetic Causality and Potential Mediators of Periodontal Disease and Atrial Fibrillation

**DOI:** 10.1016/j.identj.2024.12.029

**Published:** 2025-02-22

**Authors:** Xiaohan Zhang, Chengzhong Lian, Shuqing Shi, Jiaran Li, Lianxin Wang, Zezhen Guo, Naixu Liu, Huan Wang, Yuanhui Hu, Bai Du

**Affiliations:** a Department of Cardiovascular Medicine, Guang'anmen Hospital, China Academy of Chinese Medical Sciences, Beijing, China; b Department of Oral Medicine, Shanxi Provincial People's Hospital, Taiyuan, Shanxi, China; c Institute of Basic Research in Clinical Medicine, China Academy of Chinese Medical Sciences, Beijing, China; d Faculty of Medicine, Health and Human Sciences, Macquarie University, Sydney, New South Wales, Australia; e Department of Pediatrics, Affiliated Hospital of Nanjing University of Chinese Medicine, Nanjing, China

**Keywords:** Periodontal disease1, Atrial fibrillation2, Cytokines3, Mendelian randomisation4, Mediating pathway5

## Abstract

**Introduction and Aims:**

This study aims to examine the possible causal link between periodontal diseases and atrial fibrillation (AF), with a focus on the modifiable risk factors that facilitate this connection.

**Method:**

Firstly, bidirectional and multivariable Mendelian Randomisation (MR) analyses were conducted using genome-wide association studies (GWAS) data on periodontal disease (87,497 cases/259,234 controls) from FinnGen and AF (55,114 cases/482,295 controls) from AFGen. Then, a 2-step MR approach was employed to evaluate the mediating role and proportions of 25 candidate factors among the direct causality between periodontal disease and AF.

**Results:**

Periodontal disease was found to be associated with an increased risk of AF (odds ratio 1.16, 95% CI 1.027-1.314, *p* = .017), independent of other covariates such as dental caries, pulp, and periapical diseases. Conversely, no causal relationship was detected indicating that AF leads to periodontal disease condition. Furthermore, in the 2-step MR analysis, 5 out of 25 candidate mediators were screened as statistically significant. Ranked by partial mediation proportion, these modifiable mediators included weight (30.3%), IL-17 (17.2%), TNF (14.08%), coronary atherosclerosis (13.4%), and hypertension (11.6%).

**Conclusion:**

Our findings demonstrated the genetic causality between periodontal disease and AF. Maintaining oral hygiene, adopting standardised periodontal therapy, and restricting body weight are critical goals for patients with periodontal disease to mitigate disease progression to AF.

## Graphical abstract

**Figure:**
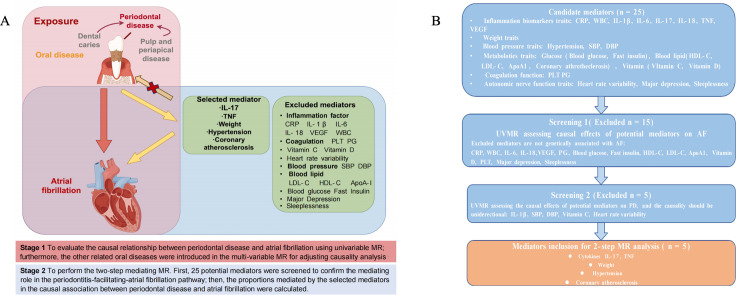

## Introduction

Periodontal disease, a chronic inflammation affecting multiple systems, is initiated by the maladjusted microbiome that progressively drives immune-cell-mediated self-degradation of the tooth-supporting apparatus and leads to tooth loss.^1^^,^^2^ With a prevalence of 50% in the adult population and 10% suffering from severe periodontitis, it stands as a neglected public health concern.^3^ Atrial fibrillation (AF), a common type of arrhythmia, manifests clinically as paroxysmal palpitation, chest tightness, and exhaustion.^4^ It is estimated that the global prevalence of AF was 50 million in 2020, the lifetime prevalence is around 30% in European and American populations, and about 20% in African American populations.^5^^,^^6^ However, it remained elusive in comprehending its underlying causes and devising effective preventive therapies.^7^ We hypothesised the biological plausibility that chronic oral infection initiates the release of bacteria and their products into the bloodstream, triggering the host inflammatory response, thereby promoting the formation and exacerbation of AF.^8^^,^^9^

Extensive epidemiological studies have investigated the detrimental systemic effects of periodontal disease beyond oral health. Recently, some cohort trials have demonstrated that periodontal disease is associated with an increased risk of AF morbidity with adjusted hazard ratio (HR) of 1.31 (95% CI, 1.06-1.62) and postoperative recurrence with odds ratio (OR) of 1.937 (95% CI, 1.301-2.884).^10^, ^11^, ^12^ Nonetheless, the correlation remains inconclusive for the reported contrasting results.^13^ This highlights the need for further research to elucidate the precise mechanisms linking periodontal disease to AF and to determine the potential benefits of periodontal treatment in reducing AF incidence and recurrence. It is plausible that cardiac arrhythmias could potentially be affected by systemic inflammation, concomitant with oral inflammation and/or by autoimmunity in the heart caused by the host immune response to specific oral pathogens.^14^^,^^15^ Moreover, the stimulation of the autonomic nervous system (ANS) and metabolic dysfunction triggered by oral pathogenic microorganisms may also contribute to arrhythmic responses.^16^^,^^17^ Subsequent literature screening of the above pathological mechanisms revealed 25 mediating factors validated by clinical investigations.

Mendelian randomisation (MR) is a causal inference technique that utilises instrumental variants (IVs) in genetic variation as proxies for exposures, akin to conducting a natural randomised controlled trial.^18^ This approach addresses observational study limitations like confounding effects and reverse causation. Through bidirectional and replicated MR analyses, the causation between periodontal disease and AF can be assessed effectively. Besides, to appraise the link of multiple risk factors with the outcome and explore the mediating effects, the traditional 2-sample MR method was extended.^19^ Multivariable MR (MVMR) and the 2-step MR were applied in this study simultaneously. Therefore, the independent causality between periodontal disease and AF was investigated, particularly in evaluating the mediating roles of inflammation factors, metabolites, and other mediators in the pathogenesis of periodontal disease-facilitating AF.

## Materials and methods

### Study design

**Fig. 1:**
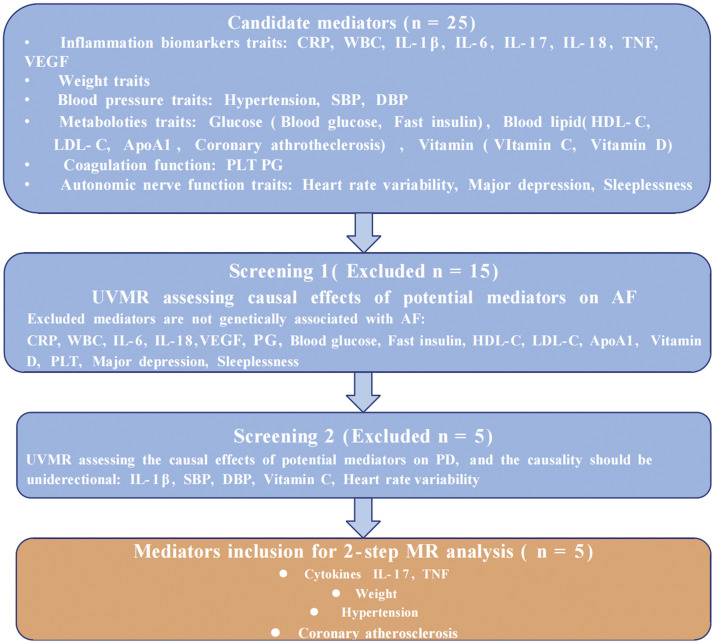
The flowchart and standard of inclusion for mediators screening. ApoA-I, apolipoprotein A-I; CRP, C-reactive protein; DBP, diastolic blood pressure; PG, prostaglandin; HDL-C, high-density lipoprotein-cholesterol; IL, interleukin; LDL-C, low-density lipoprotein-cholesterol; MR, Mendelian randomisation; PLT, platelets; SBP, systolic blood pressure; TNF, tumour necrosis factor; VEGF, vascular endothelial growth factor; WBC, white blood cell

This study consists of 2 primary stages, as depicted in Figure 1. In the first stage, 2-sample bidirectional univariate Mendelian randomisation (UVMR) and MVMR were conducted, adjusting for other common dental conditions, to examine the independent causal association between periodontal disease and AF. In the second stage, 25 candidate mediators potentially involved in the pathway linking periodontal disease to AF will be identified and screened, followed by a 2-step MR analysis to verify each partial mediating effect.

This study rigorously adheres to the 3 elementary assumptions of MR: (1) there is a robust association between IVs and the exposure (relevance); (2) IVs affect the outcome merely through the exposure (exclusion restriction); and (3) the genetic variants are not associated with any confounders of the exposure-outcome relationship (exchangeability).^20^

### Data sources and IVs selection

In Table 1, the data sources and the selection of exposure factors, mediators, and outcomes are itemised, which was primarily conducted on European ancestry. To meet the first MR assumption, single nucleotide polymorphisms (SNPs) highly correlated with the exposure were selected, with a threshold of *P* value of 5 ✕ 10^−6^ for periodontal disease and 5 ✕ 10^−8^ for AF. Then, the linkage disequilibrium (LD) test was used to identify independent SNPs, as the clumping criteria were set up as r^2^ < 0.001 and a physical distance of 10,000 kb. Subsequently, all retained SNPs related to exposure were screened on the Phenoscanner website (http://www.phenoscanner.medschl.cam.ac.uk/) to testify whether the presence or absence of confounders.^21^

**Table 1:** The summary of the GWAS database used for MR analysis.

Phenotype	Sample size	Ancestry	Consortium	Publication Year	Unit	PMID/GWAS id
	(case/control)					
**Exposure**						
Periodontitis	87497/259234	European	FinnGen	2023	logOR	finngen_R9_K11_GINGIVITIS_PERIODONTAL
Periodontitis	4544/6120	European	ARIC	2016	logOR	26962152
Dental caries	25095/352182	European	FinnGen	2023	logOR	K11_CARIES_2
Oral mucosa disease	4895/259234	European	FinnGen	2023	logOR	K11_LIP_ORAL_ MUCOSA
Pulp and periapical disease	12078/259234	European	FinnGen	2023	logOR	K11_PULP_PERIAPICAL
**Outcome**						
Atrial fibrillation	55114/482295	European	AFGen + Broad AF	2018	Event	29892015
**Included candidate mediators**						
IL-17	3301	European	INTERVAL	2018	SD	30206230
TNF	3301	European	INTERVAL	2018	SD	29875488
Hypertension	119731/343202	European	MRC-IEU	2018	Event	ukb-b-14057
Weight	461632	European	MRC-IEU	2018	SD	ukb-b-11842
Coronary atherosclerosis	14334/346860	European	Neale lab	2018	Event	ukb-d-I9_CORATHER
**Excluded candidate mediators**						
CRP	61308	European	Within family GWAS	2021	SD	ieu-b-4764
IL-1β	8293	European	Young Finns Study	2022	SD	27989323
IL-6	8293	European	Young Finns Study	2022	SD	27989323
IL-18	8293	European	Young Finns Study	2022	SD	27989323
VEGF	8293	European	Young Finns Study	2022	SD	27989323
WBC	563946	European	Blood Cell	2020	SD	ieu-b-30
PLT	166066	European	UK Biobank + INTERVAL	2016	SD	27863252
PG	10708	European	Fenland	2020	SD	33328453
SBP	757601	European	International Consortium of Blood Pressure	2018	SD	30224653
DBP	757601	European	International Consortium of Blood Pressure	2018	SD	30224653
Vitamin C	64979	European	MRC-IEU	2018	SD	ukb-b-19390
Vitamin D	496946	European	MRC-IEU	2020	SD	32242144
SBP	757601	European	International Consortium of Blood Pressure	2018	SD	30224653
DBP	757601	European	International Consortium of Blood Pressure	2018	SD	30224653
LDL-C	440546	European	UK Biobank	2020	SD	32203549
HDL-C	440546	European	UK Biobank	2020	SD	32203549
Apolipoprotein A	20687	European	FINNRISK	2016	SD	27005778
Blood glucose	58074	European	MAGIC	2012	SD	22581228
Fast Insulin	108557	European	MAGIC	2012	SD	22885924
Heart rate variability	27850	European	Meta	2017	SD	28613276
Major Depression	170756/329443	European	PGC	2019	Event	30718901
Sleeplessness	462341	European	MRC-IEU	2018	SD	ukb-b-3957

ApoA-I, apolipoprotein A-I; CRP, C-reactive protein; DBP, diastolic blood pressure; PG, prostaglandin; FDR, false discovery rate; HDL-C, high-density lipoprotein cholesterol; IL, interleukin; IVW, inverse variance weighted; LDL-C, low-density lipoprotein cholesterol; MR, Mendelian randomization; PLT, platelets; SBP, systolic blood pressure; TNF, tumour necrosis factor; VEGF, vascular endothelial growth factor; WBC, white blood cell.

#### Exposure

For the discovery cohort, IVs for exposure were obtained from the IEU Open GWAS project (https://gwas.mrcieu.ac.uk/), specifically utilizing the GWAS data from the dental cohort of the Atherosclerosis Risk in Communities (ARIC) Study.^22^ Participants were briefly grouped into periodontal health (PPC-A), mild PD (PPC-B and C), moderate PD (PPC-D and E), and severe PD (PPC-F and G), with severe PD phenotype was included in this MR study.

For replication, SNPs associated with periodontal disease were extracted from the largest and most recently released FinnGen R9 project, a Finnish nationwide GWAS linked with longitudinal phenotype and digital health records.^23^ This consortium included 259,234 controls and 87,497 individuals with periodontal disease (including gingivitis and periodontitis). Following retrieval from the Phenoscanner database, several SNPs were excluded due to associations with confounding factors: rs17045199 for pulse rate and red cell distribution width, rs17759178 for basal metabolic rate and whole-body water/fat-free mass, rs9490847 due to its pleiotropic association with hypertension,^24^ and rs117806480 for its association with coronary heart disease.^25^ This dataset was subsequently used in the MVMR and 2-step mediating MR analyses.

#### Outcomes

IVs for AF were selected from the largest published meta-analysis GWAS including more than 50 studies, mainly covered by the Atrial Fibrillation Genetics (AFGen) consortium and the Broad AF Study with a total of 97 distinct AF loci from 65,446 cases and more than 522,000 controls.^26^

#### Mediators

Based on the literature review, we identified 25 candidate mediators that may increase the susceptibility to AF induced by periodontal disease (shown in Figure 1 and Table S1) as confirmed by clinical investigations. They comprise cytokines, inflammatory factors, metabolic-related indicators, and indices reflecting autonomic nervous function.^27^, ^28^, ^29^, ^30^, ^31^, ^32^, ^33^, ^34^, ^35^, ^36^, ^37^, ^38^, ^39^, ^40^, ^41^, ^42^, ^43^, ^44^, ^45^, ^46^, ^47^, ^48^, ^49^, ^50^ The summary of the detailed GWAS dataset selection is itemised in Table 1.

This study complied with the following criteria in determining the included mediators: (1) A causality between periodontal disease and a mediator exists, which is supposed to be unidirectional^51^; (2) The causality consistently exists between the mediator and AF; (3) Based on current literature foundation, the causality of periodontal disease and mediators, as well as mediators and AF, should be in the same directions.

### Statistical analysis

#### UVMR and MVMR analysis

The TwoSample MR package in RStudio was employed for this study. Causality was primarily evaluated using random-effects inverse variance weighting (IVW) and the average weighted derivative of Wald ratio estimates. Results were subsequently converted into OR values for binary variables. Multivariable IVW analyses were used to detangle and compensate for the impact of gene-phenotype relationships with other oral disorders, comparing the causal link between periodontal disease and AF.

To mitigate the influence of weak instrument bias, a critical assumption in MR analysis, we assessed the strength of selected genetic IVs using F statistics, where values above 10 indicate robustness.^52^^,^^53^ While assuming validity of all IVs, the IVW method inherently possesses limitations, addressed through MR sensitivity analyses. The MR-Egger method and weighted median model were employed to test the null causal hypothesis and provide reliable causal effects amidst evident pleiotropy.^54^^,^^55^ To detect bias related to heterogeneity, the MR pleiotropy residual sum and outlier (MR-PRESSO) R package was utilised (Nb distribution = 10,000, significant threshold = 0.05), alongside a leave-one-out SNP analysis to identify outliers. A significance threshold of *p* < .05 was set for causal effects and sensitivity analyses. Considering type I error, sample overlap estimates in discovery and replication analyses (24.8% and 6.0%) were calculated using the "Overlap" R package and further bias was assessed using an online tool^11^^,^^56^ (Table S6).

#### Mediation MR analysis

The current study employed a 2-step MR analysis to investigate the intermediary role of mediators in the pathogenesis of periodontal disease facilitating AF. Initially, 25 mediators associated with AF and supported by strong evidence were evaluated using UVMR to determine their genetic causality on AF. Subsequently, the unidirectional causation of periodontal disease on these included mediators was assessed through an additional UVMR.

To control for multiple comparisons, the Benjamini-Hochberg method was applied to obtain adjusted q-values for false discovery rate (FDR) correction. Results from the IVW analysis with a *p*-value < .05 and FDR q-value < 0.05 were considered strong evidence, while those with a *p*-value < .05 and FDR q-value ≥ 0.05 were categorised as indicative evidence.^57^ Only mediators demonstrating causality with periodontal disease, which subsequently influenced the risk of AF, were included in further analysis. To quantify the partial mediating proportion, the mediating effect was calculated as the product (β1 * β2) of the exposure on each mediator (β1) and the direct effect of mediators on AF adjusting for periodontal disease (β2), divided by the total effect. Standard errors (SE) were derived using the delta method.^55^

## Results

### Univariable and multivariable MR analysis for assessing the causality of periodontal disease on AF

**Fig. 2:**
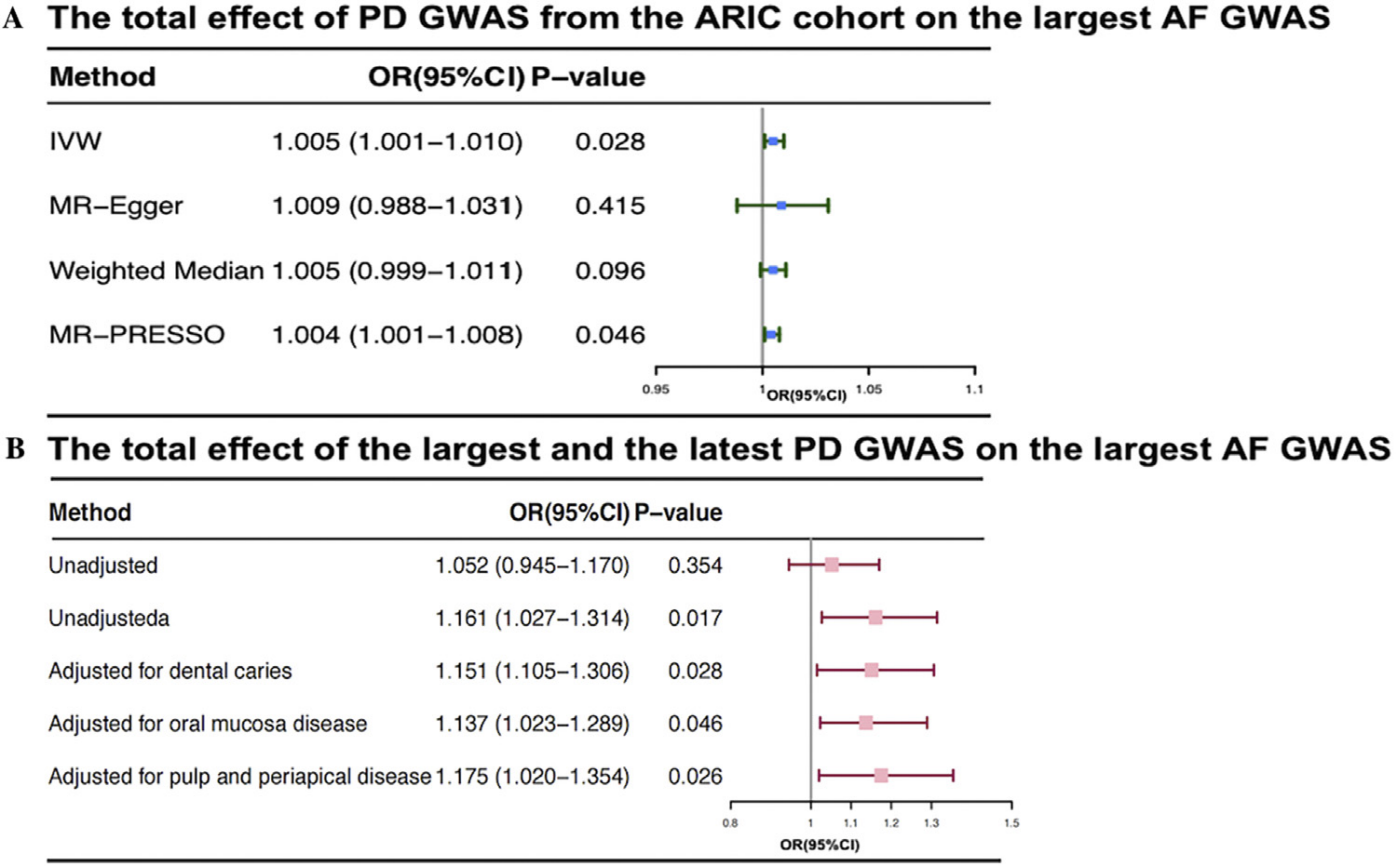
The summarised results of univariable and multivariable MR analysis for assessing the independent causality of periodontal disease on AF. (A) Total effect of PD on AF assessed by the discovery MR analysis using PD GWAS from the ARIC cohort; (B) Total effect of PD on AF validating MR results using the latest, the largest and non-overlapping NAFLD GWAS from the FinnGen. OR (95% CI) represents per SD increases of PD associated with the risk of AF; "Unajusted^a^" represents the MR analysis after removing potential confounding factors. AF, atrial fibrillation; ARIC, atherosclerosis risk in communities; CI, confidence interval; GWAS, Genome-Wide Association Study; IVW, inverse variance weighting; MR-PRESSO, Mendelian randomisation-pleiotropy residual sum and outlier; OR, odds ratio.

In the UVMR analysis for discovery (Figure 2A), individuals with periodontal disease showed a slightly increased risk of AF by 0.5% (OR 1.005, 95% CI, 1.001-1.01; *P* = .028). Due to limitations in sample size, the UVMR findings were subsequently replicated using the largest and most recent FinnGen GWAS database for exposure to confirm causality. Addressing horizontal pleiotropy observed in the initial validation analysis, which indicated inconsistent results across various statistical methods, confounding factors were rigorously controlled. This validation demonstrated a positive causal effect, with an OR for periodontal disease of 1.16 (95% CI: 1.03-1.31; *P* = .017) per 1-unit log odds increase in AF risk.

Conversely, both discovery and validation analyses found insufficient evidence to support a positive genetic prediction of AF impacting periodontal disease. (see Table S4). The validity was confirmed through the MR-Egger intercept and Cochran's Q tests, which indicated no significant pleiotropy or heterogeneity in each MR analysis (see Table S5).

In the subsequent MVMR analysis (Figure 2B), the causal relationship between periodontal disease and AF remained positive even after adjusting for potential confounding factors such as dental caries (IVW OR: 1.15, 95% CI: 1.105-1.306), oral mucosa disease (IVW OR: 1.14, 95% CI: 1.223-1.289), and pulp and periapical disease (IVW OR: 1.17, 95% CI: 1.02-1.35). All directions of IVW results in MVMR were consistent with those of MVMR Egger sensitivity analyses results. Moreover, assessments of horizontal pleiotropy and heterogeneity indicated a low probability, as suggested by Cochran's Q statistic and Egger intercept values (see Table S7).

### The 2-step MR analysis for exploring the mediating factors in the impact of periodontal disease on AF promotion

#### UVMR analysis for the causality of 25 potential mediators on AF

**Fig. 3:**
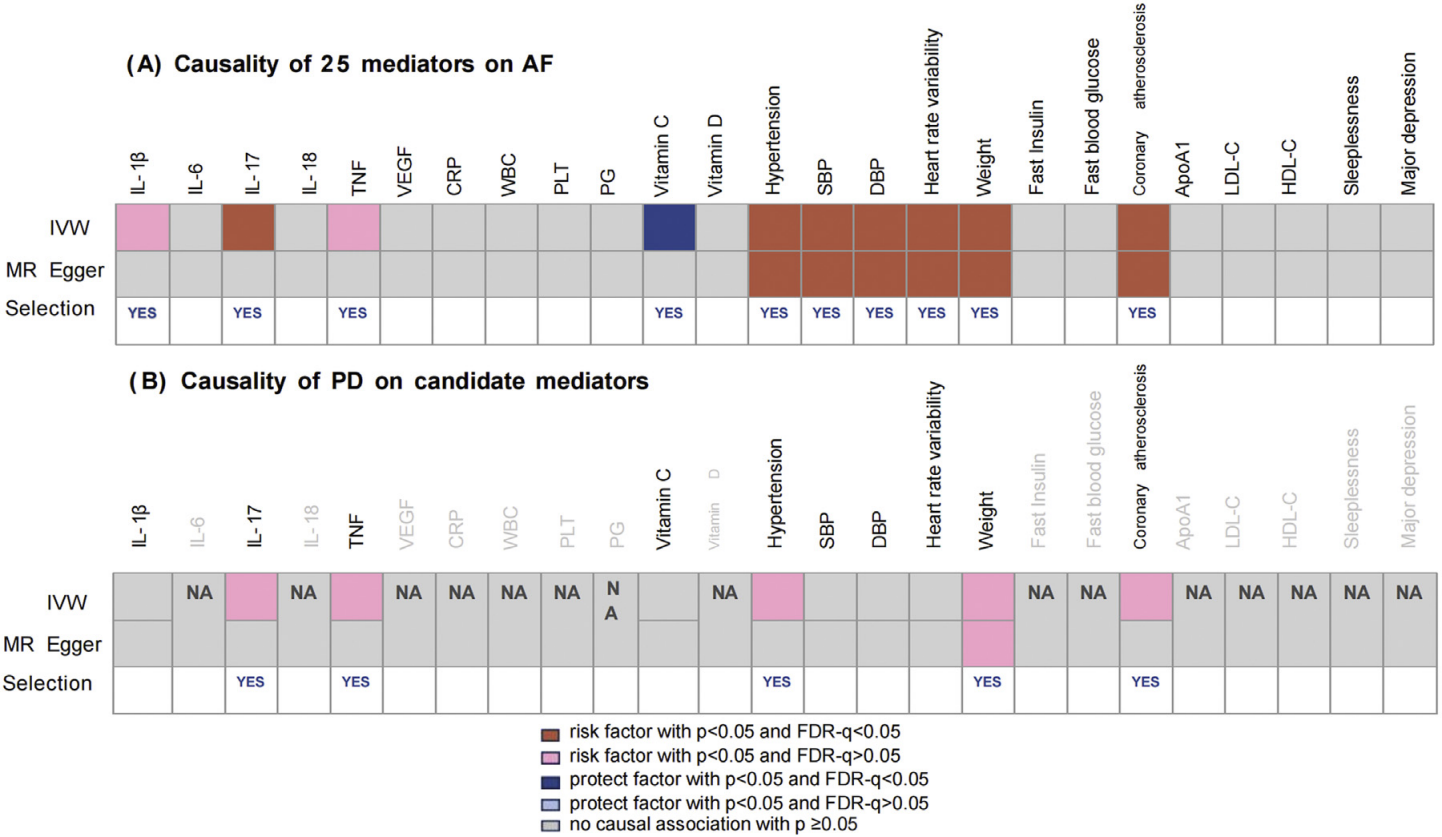
Evidence grade for mediators’ selection in the association of PD on AF. (A) Causality of potential mediators on AF; (B) Causality of PD on candidate mediators which were proved to genetically influence AF. The IVW result was the main standard for mediators’ inclusion. Marking "YES" in the "Selection" column represents the candidate mediators selected for the next step analysis. ApoA-I, apolipoprotein A-I; CRP, C-reactive protein; DBP, diastolic blood pressure; PG, prostaglandin; FDR, false discovery rate; HDL-C, high-density lipoprotein cholesterol; IL, interleukin; IVW, inverse variance weighted; LDL-C, low-density lipoprotein cholesterol; MR, Mendelian randomisation; PLT, platelets; SBP, systolic blood pressure; TNF, tumour necrosis factor; VEGF, vascular endothelial growth factor; WBC, white blood cell; WM, weighted median.

As illustrated in Figure 3, 10 of 25 candidate mediator exhibited a positive relationship with the risk of AF. Specifically, 8 mediators showed strong evidence of genetic correlation with AF risk after FDR correction: IL-17 (IVW β: 0.047 SD; 95% CI: 0.014-0.08), Vitamin C (IVW β: -0.264 SD; 95% CI: -0.472 to -0.056), hypertension (IVW OR: 2.756 SD; 95% CI: 2.273-3.342), systolic blood pressure (SBP) (IVW β: 0.015 SD; 95% CI: 0.011-0.019), diastolic blood pressure (DBP) (IVW β: 0.023 SD; 95% CI: 0.015-0.031), heart rate variability (IVW β: 0.276 SD; 95% CI: 0.086-0.466), weight (IVW β: 0.5 SD; 95% CI: 0.437-0.563), and coronary atherosclerosis (IVW OR: 3.565 SD; 95% CI: 1.50-8.472). Additionally, IL-1β (IVW β: 0.06 SD; 95% CI: 0.005-0.028) and TNF (IVW β: 0.04 SD; 95% CI: 0.001-0.079) were selected for further analysis based on indicative evidence. To ensure an adequate number of SNPs, the *P* value threshold for SNP selection was relaxed to 5 ✕ 10^−6^; none of these SNPs exhibited weak instrumental variable bias after F-statistic testing (see Table S3). As suggested by the MR-Egger intercept and Cochran's Q results, the horizontal pleiotropy was denied while the heterogeneity from IVs was inevitable (Table S9).

#### UVMR analysis for the causality of periodontal disease on included mediators

Out of the 10 screened mediating factors associated with AF risk, 5 mediators are likely to be genetically determined by suffering from periodontal disease (Figure 3). Those were IL-17 (IVW β: 0.429; [95% CI: 0.059-0.799]), TNF (IVW β: 0.429 SD; [95% CI: 0.0586-0.896]), hypertension (IVW β: 0.017 SD; [95% CI: 0.001-0.033]), weight (IVW β: 0.091; [95% CI: 0.015-0.167]), coronary atherosclerosis (IVW β: 0.006 SD; [95% CI:0.001-0.012]), respectively. In contrast, the other 5 mediators were unable to present the genetically influenced by periodontal disease. Heterogeneity among IVs seems to be ineluctable due to the large number of SNPs, whereas the rejection of horizontal pleiotropy suggested by the MR-Egger intercept was definite (Table S11).

#### Two-step MR analysis for evaluating the mediating effects between periodontal disease and AF

**Fig. 4:**
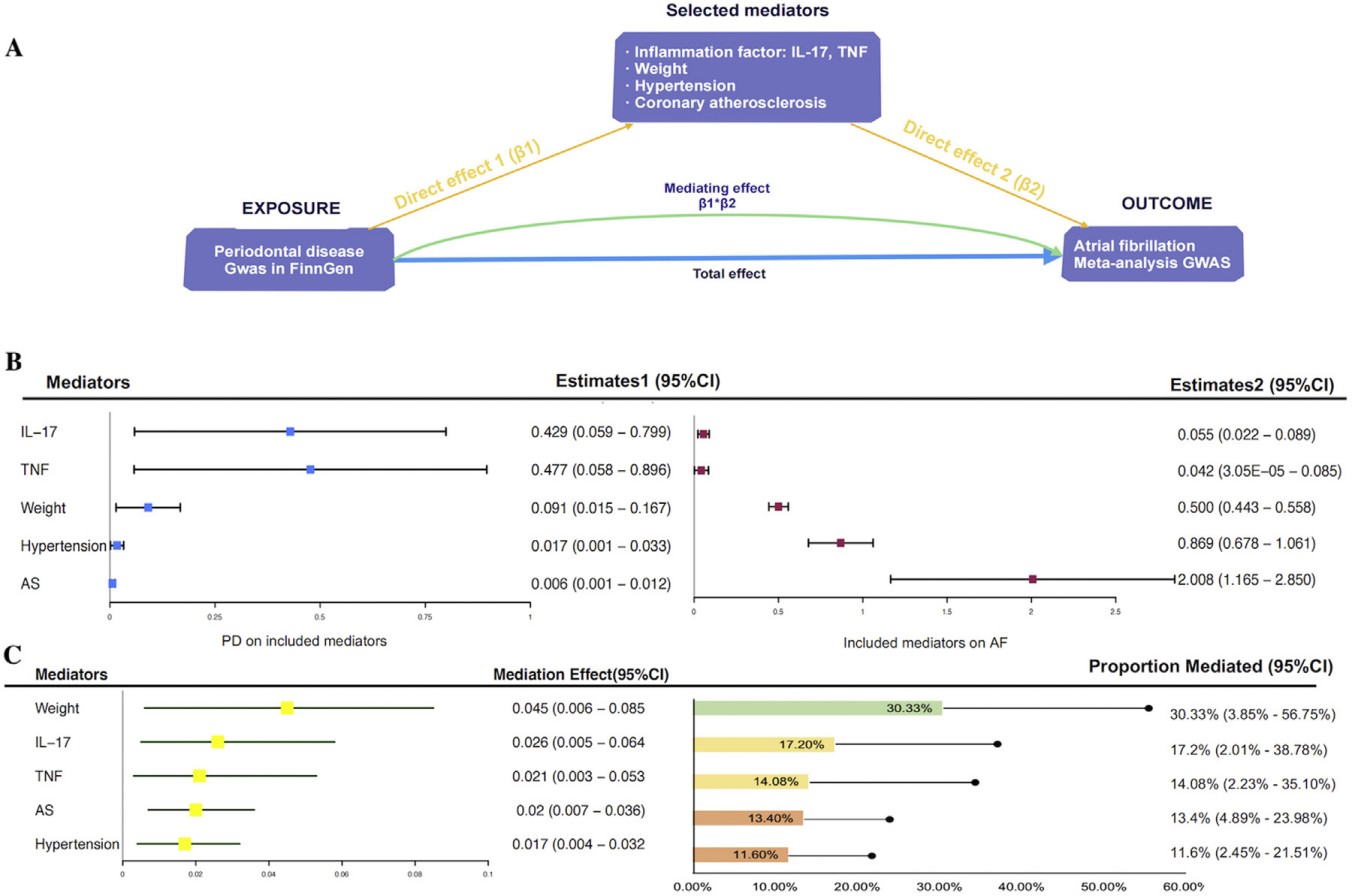
The 2-step MR analysis for investigating the mediating pathway between PD on AF. (A) The framework of the 2-step MR analysis; (B) The visually summarised MR results between mediators on exposure and outcome. The left part is the causal effects of PD on mediators (β1) and the right part is the causal effects of mediators on AF with adjustment for PD (β2); (C) The mediating effects of the ultimately included mediators (left) and their respectively mediating proportion (right). CI, confidence interval; GWAS, Genome-Wide Association Study; IL, interleukin; AS, Atherosclerosis; TNF, tumour necrosis factor.

Following investigation via 2 UVMR analyses, IL-17, TNF, hypertension, body weight, and coronary atherosclerosis were selected for calculating their mediating effects (Figure 4). These 5 mediators were genetically influenced by periodontal disease and maintained a positive causality with AF even after adjustment for periodontal disease (Table S12). Body weight exhibited the highest mediation at 30.3% (95% CI: 3.85%-56.75%), followed by IL-17 (17.2%; 95% CI: 2.01%-38.78%), TNF (14.08%; 95% CI: 2.23%-35.10%), coronary atherosclerosis (13.4%; 95% CI: 4.89%-23.98%), and hypertension (11.6%; 95% CI: 2.45%-21.51%).

**Fig. 5:**
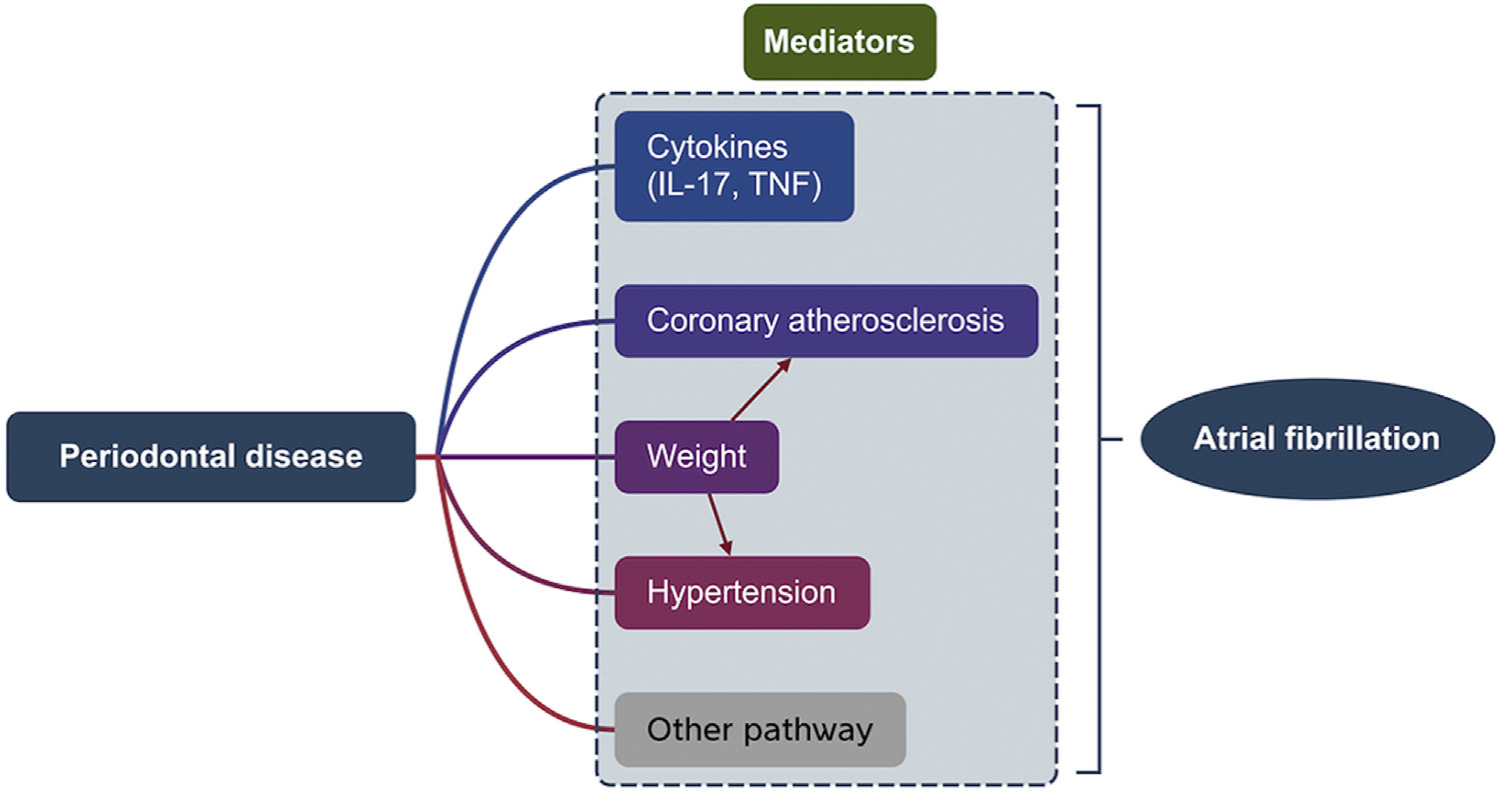
The directed acyclic diagram shows the interaction among the 5 selected mediators amid pathways of PD- PD-triggering- AF. The colourful modules represent the mediators demonstrated by this study. The red arrows indicate causality between weight and other mediators such as hypertension and coronary atherosclerosis, with MR estimates attached in Table S13. The grey module represents other potential mediating pathways that need further investigation connecting PD and AF. IL, interleukin; TNF, tumour necrosis factor.

Probing into the interactive relationship between weight, the most prominent mediator, and other selected mediators (Figure 5), a bidirectional MR was performed. Higher weight is genetically linked to hypertension and coronary atherosclerosis, which may further aggravate periodontal disease and AF promotion. No significant result was observed in the inverse MR; however, more candidate mediating pathways still need to be analyzed.

## Discussion

This MR study evaluated the association between periodontal disease and increased risk of AF, yielding an OR of 1.16 accounting for genetic predisposition. This positive causal relationship remained significant even after adjusting for dental caries and pulp diseases. Subsequent mediation analysis identified 5 potential mediators, with weight contributing the most at 30.3%, followed by IL-17 (17.2%), TNF (14.08%), coronary atherosclerosis (13.4%), and hypertension (11.6%). At the molecular level, preliminary evidence suggests that inflammation and immune responses mediated by cytokines may be key pathological factors linking periodontal disease to heightened AF risk, emphasizing weight management as a viable preventive treatment.

Poor oral hygiene, a prevalent public health concern affecting a substantial population, has been linked to temporary bacteremia and chronic low-graded inflammation, an immunological response associated with cardiovascular disease pathogenesis.^58^ A cohort in Japan histologically revealed that periodontal inflammation surface area was positively correlated with atrial fibrosis (R = 0.46, *P* < .0001) and remained significant (β = 0.016, *P* = .0002) even after adjusting for multiple covariates.^59^ Similarly, another dental cohort reported that severe periodontitis was associated with AF (adjusted HR: 1.31; [95% CI, 1.06-1.62]), correspondingly, compared with episodically dental care users, regular users had a lower risk for AF (adjusted HR: 0.88, [95% CI, 0.78-0.99]); and AF mediates the association between periodontal disease and stroke.^11^ In contrast, the current MR study did not provide evidence for AF being genetically influenced by periodontal disease.^14^ Our research developed the previous study by integrating the largest and the most recent periodontal disease GWAS and the largest AF GWAS in the 2-sample MR, with results that aligned with earlier observational studies and corroborated the genetic liability hypothesis while discarding environmental confounders. Additionally, the study examined mediators involved in the causal relationship from periodontal disease to AF, contributing to up-to-date studies.

The hypothesised mechanism linking periodontal disease to AF mainly focused on systemic inflammation driven by dental plaque and the local release of cytokines. Low-level bacteraemia may enter the bloodstream, enabling heart invasion and triggering autoimmunity against cardiac molecular structures due to the host's immune response to specific oral pathogens.^60^ Furthermore, chronic inflammation is also thought to overactivate the ANS, resulting in electrical remodelling in AF.^61^

Drawing upon the existing knowledge base and high-quality GWAS database, we picked out 25 mediators that were relevant to intermediate pathways with definite epidemiological evidence. Consistent with current scientific findings, cytokines-mediated immune response and systemic inflammation are considerably important intermediate factors in promoting the development of AF among periodontal disease patients.^30^^,^^31^ Overweight was identified as the most prominent mediator, which may contradict clinical perceptions, given that severe periodontal disease can result in loss of appetite and weight. Some studies revealed the non-linear correlation of mild-to-moderate periodontitis on obesity which is presumably out of the disruption in the cytokines network triggered by periodontal bacteria and their virulence factors such as the lipopolysaccharide.^62^^,^^63^ This pathway can be attributed to a sharing mechanism among mediators. The interaction between weight and the biological pathways of immune-inflammatory activation, energy metabolism, and neuroendocrine regulation establishes it as the most potent mediator.^64^ For instance, as the release of cytokines acts in both autocrine and paracrine manners in various cells of metabolic tissues and contributes to metabolic disorders, low-grade inflammation leads to fat accumulation, dyslipidaemia, and insulin resistance (IR) with the elevation of blood glucose.^65^^,^^66^ To disentangle the interactive causal association, this study conducted bidirectional MR analyses of weight and other selected mediators. Hypertension and coronary atherosclerosis were found to be genetically determined by overweight, while the inversed causality of other mediators on weight was not significant.

The discrepancy between MR results and observational studies is objective. On the one hand, observational studies are subject to external environmental confounding and reversed causal bias. Although the role of various inflammation factors (i.e., CRP, IL-1β, IL-6, etc.) and metabolic factors (i.e., blood glucose, IR, blood lipid, etc.) had been verified by multiple compelling clinical investigations,^26^, ^27^, ^28^, ^29^^,^^34^^,^^42^^,^^43^ these findings were not corroborated by genetic causality in the UVMR. Notably, CRP, IL-6, along with other cytokines, elevated in response to oral bacteria entering the bloodstream, triggering systemic inflammation, have been identified as genetic loci positively correlating with atrial remodelling and AF, and biomarkers of disease status.^67^, ^68^ Through mediation analysis, we further delineated TNF and IL-17 as causal mediators linking periodontal disease and AF intervention and prevention, while other inflammatory factors appeared to be uninvolved in pathogenesis and merely served as indicators of the progression. On the other hand, despite passing the F statistic test, the insufficient power due to low variability of extracted IVs could remain bias in the MR analysis and yield inconsistencies with real-world studies.

As far as our concern, this is the very first MR study to demonstrate the positive causal association between periodontal disease and AF independently. Then, we screened and validated candidate mediating pathways. There are several strengths of our study. First, we utilised 2 GWAS datasets for periodontal disease and the largest-scale GWAS for AF in UVMR, ensuring precise causal predictions. Secondly, several sensitivity analyses were complemented to confirm the robustness of IVW results. Thirdly, rigorous adherence to MR analytic protocols minimised confounding and reverse causation, thereby ensuring the reliability of our causal model illustrating the association and mediating pathways. Fourthly, rigorous criteria for mediator screening were set to reduce the reverse causation of mediators on periodontal disease and ensure the credibility of the model constructed for the partial mediating effect in the 2-step MR. Clarifying mediators within the causal pathway between periodontal disease and AF helps differentiate them from biomarkers, facilitates the selection of prevention targets, and offers a foundation for future basic research.

Admittedly, this study also had some limitations. First, in our analysis, we assumed a linear relationship between periodontal disease and AF; however, more research is needed to explore any potential non-linear associations by introducing regression analysis or other statistical models. Second, the current mediation MR model potentially restricted the comprehensive screening by focusing exclusively on the causal pathway from periodontal disease to AF, without full identification of genetic factors associated with periondontal disease, AF or their comorbidity. Moreover, mediators were mainly selected based on observational studies, which might have overlooked non-heritable factors or mediators for which GWAS data is unavailable. Third, the selected bias caused by GWAS extracted by European populations in developed countries with high oral hygiene standards, underscoring the need to validate our findings in other ethnic groups or developing countries. Fourth, to incorporate adequate SNPs for enhanced analytical quality, we adjusted the *P* value of the inclusion criteria for periodontal disease. Although previous studies suggest a threshold of 5 × 10⁻^6^ for this exposure, it may still yield weak IVs correlation.^69^

## Conclusions

This MR investigation elucidated that the presence of periodontal disease significantly elevates the incidence of AF, independent of confounding factors such as dental caries and periapical disease. Subsequently, several inflammatory factors, including IL-17 and TNF, along with associated pathological conditions such as overweight and coronary atherosclerosis, were identified as key mediators in the causal pathway from periodontal disease to AF. Notably, overweight emerged as the most prominent mediator. It is crucial to underscore the significance of oral hygiene and standardised periodontal treatment in order to prevent and intervene in AF in individuals with periodontal disease. These interventions attenuate systemic inflammation, highlighting the critical role of oral health in comprehensive AF prevention strategies.

## Ethics approval and consent to participate

Not applicable. This study used publicly available data from previous studies approved by an ethical standards committee. Therefore, no further ethical approval was required in this study.

## Author contributions

Conceptualisation: Du. and Zhang; methodology, Hu; software: Lian, Li. and Liu; validation, Shi and Guo; formal analysis, L. Wang; investigation, H. Wang; resources, Zhang.; data curation, Shi.; writing—original draft preparation, Lian and Zhang; writing—review and editing, Guo.; supervision, Du and Hu; funding acquisition, Du, Shi, and Hu. All authors have read and agreed to the published version of the manuscript.

## Funding

This work was supported by the National Natural Science Foundation of China (No. 82205096), the Scientific and Technological Innovation Project of the China Academy of Chinese Medical Sciences (No.CI2021A00918 and No. CI2021A03318), and the Central High-Level Chinese Medicine Hospital Promotion Project (No. HLCMHPP2023106).

## Conflict of Interest

None disclosed.

## Appendix Supplementary materials

Image, application 1

Image, application 2

Image, application 3

Image, application 4

## Footnotes

Supplementary material associated with this article can be found in the online version at doi:10.1016/j.identj.2024.12.029.
