# Supplementary material for: The 2-Step Mendelian Randomisation Study Assesses Genetic Causality and Potential Mediators of Periodontal Disease and Atrial Fibrillation
Source: Int Dent J. 2025 Feb 22;75(3):2093–103. doi: 10.1016/j.identj.2024.12.029 (PMC12142772; doi:10.1016/j.identj.2024.12.029)
Supplement: Supplementary file 1 [file mmc1.docx]

**STROBE-MR checklist of recommended items to address in reports of Mendelian randomization studies**^1^ ^2^

| **Item No.** | **Section** | **Checklist item** | **Page No.** | **Relevant text from manuscript** |
| --- | --- | --- | --- | --- |
| 1 | **TITLE and ABSTRACT** | Indicate Mendelian randomization (MR) as the study’s design in the title and/or the abstract if that is a main purpose of the study | 1 | Causality and Modifiable Risk Factors of Periodontal Disease and Atrial Fibrillation Assessed by Two-Step Mendelian Randomization Study |
|  | **INTRODUCTION** |  |  |  |
| 2 | **Background** | Explain the scientific background and rationale for the reported study. What is the exposure? Is a potential causal relationship between exposure and outcome plausible? Justify why MR is a helpful method to address the study question | 4 | Recently, some cohort trials have demonstrated that periodontal disease is associated with an increased risk of AF morbidity and recurrence. Mendelian Randomization is a causal inference method that exploits instrumental variants (IVs) as a proxy for exposure, which resembles conducting a natural randomized control trial |
| 3 | **Objectives** | State specific objectives clearly, including pre-specified causal hypotheses (if any). State that MR is a method that, under specific assumptions, intends to estimate causal effects | 5-6 | We speculate the biological plausibility that the chronic oral infection leads to the release of bacteria/their products into the bloodstream, then activates the host inflammatory response, which favors the formation and exacerbation of AF. We perform the bidirectional and replicated MR analysis to evaluate the causation between periodontal disease and AF. Besides, to appraise the link of multiple risk factors with the outcome and explore the mediating effects, the traditional 2-sample MR method was extended. Multivariable MR (MVMR) and two-step MR were applied in this study simultaneously. Therefore, independent causality between periodontal disease and AF was investigated, particularly in evaluating the mediating effects of inflammation factors, metabolites and other mediators concerning the pathogenesis of periodontal disease-facilitating AF. |
|  | **METHODS** |  |  |  |
| 4 | **Study design and data sources** | Present key elements of the study design early in the article. Consider including a table listing sources of data for all phases of the study. For each data source contributing to the analysis, describe the following: |  |  |
|  | a) | Setting: Describe the study design and the underlying population, if possible. Describe the setting, locations, and relevant dates, including periods of recruitment, exposure, follow-up, and data collection, when available. | 5 | First, the two-sample bidirectional univariate MR (UVMR) and MVMR adjusted by other common oral diseases will be performed to explore the independent causality of periodontal disease and AF. Secondly, we selected and screened 25 candidate mediators in the periodontal disease-facilitating AF pathway and then resorted to the two-step MR analysis to confirm each mediating effect. |
|  | b) | Participants: Give the eligibility criteria, and the sources and methods of selection of participants. Report the sample size, and whether any power or sample size calculations were carried out prior to the main analysis | 7-8 | We extracted SNPs for periodontal disease from the largest and the newly released FinnGen R9 project, a Finnish nationwide GWAS linked with longitudinal phenotype and digital health records[17]. This consortium included 259234 controls and 87497 individuals with periodontal disease (gingivitis and periodontitis).  IVs for AF were selected from the largest published meta-analysis GWAS including more than 50 studies, mainly covered by the Atrial Fibrillation Genetics (AFGen) consortium and the Broad AF Study with a total of 97 distinct AF loci from 65,446 cases and more than 522,000 controls |
|  | c) | Describe measurement, quality control and selection of genetic variants | 6-7 | To meet the first MR assumption, single nucleotide polymorphisms (SNPs) were most correlated with exposure, with a threshold of P value of 5 ✕ 10-6 for periodontal disease and 5 ✕ 10-8 for AF. Then, the linkage disequilibrium (LD) test was used to identify independent SNPs, as the clumping criteria were set up as r2 < 0.001 and a physical distance of 10,000 kb. Subsequently, all retained SNPs related to exposure were screened on the Phenoscanner website to testify whether the presence or absence of confounders |
|  | d) | For each exposure, outcome, and other relevant variables, describe methods of assessment and diagnostic criteria for diseases | NONE |  |
|  | e) | Provide details of ethics committee approval and participant informed consent, if relevant | 25 | Ethics approval and consent to participate: Not applicable. This study used publicly available data from previous studies approved by an ethical standards committee. Therefore, no further ethical approval was required in this study. |
| 5 | **Assumptions** | Explicitly state the three core IV assumptions for the main analysis (relevance, independence and exclusion restriction) as well assumptions for any additional or sensitivity analysis |  |  |
| 6 | **Statistical methods: main analysis** | Describe statistical methods and statistics used |  |  |
|  | a) | Describe how quantitative variables were handled in the analyses (i.e., scale, units, model) | Table S2 | Table S2 The characteristics of the selected SNPs on periodontal disease for Mendelian randomization analysis |
|  | b) | Describe how genetic variants were handled in the analyses and, if applicable, how their weights were selected | 9-10 | To circumvent the influence of weak instrument bias, namely the first assumption in MR analysis, we evaluated the strength of the selected genetic IVs by calculating F statistics with the established formula, generally, F statistics greater than 10 are considered strong. Presuming all IVs are valid, the IVW has its inevitable defects which can be addressed by the MR sensitivity analysis. The MR-Egger method and weighted median model were introduced to test the null causal hypothesis and provide a steady causal effect in the face of the evident pleiotropy. To detect the heterogeneity-related bias, we employed the MR pleiotropy residual sum and outlier (MR-PRESSO) R package (Nb Distribution =10000, Significant Threshold = 0.05) and the leave-one-out method to identify outlier SNPs. P<0.05 was set as a statistically significant threshold of causal effects and sensitivity analysis. Considering the type I error, the estimation of the potential sample overlap on discovery and replicated analysis (24.8% and 6.0%) were executed through the “Overlap” R package and the resultant bias was calculated (Table S6) based on the online tool (https://sb452.shinyapps.io/overlap/) |
|  | c) | Describe the MR estimator (e.g. two-stage least squares, Wald ratio) and related statistics. Detail the included covariates and, in case of two-sample MR, whether the same covariate set was used for adjustment in the two samples | 9 | The TwoSample MR package in RStudio was applied for this study. Causality was chiefly confirmed by the random-effects inverse variance weighting (IVW), the average weighted derivative of the Wald ratio estimates, and then converted the result into the odds ratio (OR) value for the binary variable. In order to disentangle and adjust the effects of the gene-phenotype association of other oral diseases, the multivariable IVW was conducted to compare the causation between periodontal disease and AF. |
|  | d) | Explain how missing data were addressed |  | NONE |
|  | e) | If applicable, indicate how multiple testing was addressed | 10 | Using the Benjamini-Hochberg method, an adjusted q-value was obtained for false discovery rate (FDR) correction in multiple tests. Results from the IVW analysis with a p-value < 0.05 and FDR q-value < 0.05 were deemed strong evidence, while results with a p-value < 0.05 and FDR q-value ≥ 0.05 were categorized as indicative evidence |
| 7 | **Assessment of assumptions** | Describe any methods or prior knowledge used to assess the assumptions or justify their validity | 45 | Reference 48-50 |
| 8 | **Sensitivity analyses and additional analyses** | Describe any sensitivity analyses or additional analyses performed (e.g. comparison of effect estimates from different approaches, independent replication, bias analytic techniques, validation of instruments, simulations) | 10 | Presuming all IVs are valid, the IVW has its inevitable defects which can be addressed by the MR sensitivity analysis. The MR-Egger method and weighted median model were introduced to test the null causal hypothesis and provide a steady causal effect in the face of the evident pleiotropy |
| 9 | **Software and pre-registration** |  |  |  |
|  | a) | Name statistical software and package(s), including version and settings used | 9 | The TwoSample MR package in RStudio 4.2.1 was applied for this study |
|  | b) | State whether the study protocol and details were pre-registered (as well as when and where) | None |  |
|  | **RESULTS** |  |  |  |
| 10 | **Descriptive data** |  |  |  |
|  | a) | Report the numbers of individuals at each stage of included studies and reasons for exclusion. Consider use of a flow diagram | 7 | After retrieval on the Phenoscanner database, several SNPs were excluded for the liability of being associated with other confounding factors: rs17045199 for pulse rate and red cell distribution width, rs17759178 for basal metabolic rate, whole-body water/fat-free mass. Moreover, based on the available literature, rs9490847 has been recorded the pleiotropy for hypertension; rs117806480 was associated with coronary heart disease |
|  | b) | Report summary statistics for phenotypic exposure(s), outcome(s), and other relevant variables (e.g. means, SDs, proportions) | TableS2 |  |
|  | c) | If the data sources include meta-analyses of previous studies, provide the assessments of heterogeneity across these studies | None |  |
|  | d) | For two-sample MR:  i.  Provide justification of the similarity of the genetic variant-exposure associations between the exposure and outcome samples  ii.  Provide information on the number of individuals who overlap between the exposure and outcome studies | 10 | Considering the type I error, the estimation of the potential sample overlap on discovery and replicated analysis (24.8% and 6.0%) were executed through the “Overlap” R package and the resultant bias was calculated (Table S6) based on the online tool |
| 11 | **Main results** |  |  |  |
|  | a) | Report the associations between genetic variant and exposure, and between genetic variant and outcome, preferably on an interpretable scale | 11 | In the UVMR analysis (Figure 3A) for discovery, we found that patients who seek treatment for periodontal disease slightly suffer a higher risk of AF by 0.5% (OR: 1.005, 95% confidence interval [CI]: 1.001–1.01; P = 0.028). |
|  | b) | Report MR estimates of the relationship between exposure and outcome, and the measures of uncertainty from the MR analysis, on an interpretable scale, such as odds ratio or relative risk per SD difference | 11 | In the UVMR analysis (Figure 3A) for discovery, we found that patients who seek treatment for periodontal disease slightly suffer a higher risk of AF by 0.5% (OR: 1.005, 95% confidence interval [CI]: 1.001–1.01; P = 0.028). Limited by the sample size, the UVMR was replicated with the largest and the latest FinnGen GWAS database of exposure to verify the causality. Given the presence of horizontal pleiotropy in the initial validation analysis, which is suggested by the inconsistent results from various statistical methods, confounder factors were subsequently controlled as a result. |
|  | c) | If relevant, consider translating estimates of relative risk into absolute risk for a meaningful time period | 11 | Then the positive causality was demonstrated as well with the OR for periodontal disease of 1.16 (95%CI: 1.03-1.31; P = 0.017) per 1-unit log odds increase in AF. |
|  | d) | Consider plots to visualize results (e.g. forest plot, scatterplot of associations between genetic variants and outcome versus between genetic variants and exposure) | Figure 3 |  |
| 12 | **Assessment of assumptions** |  |  |  |
|  | a) | Report the assessment of the validity of the assumptions | 12 | A low probability of horizontal pleiotropy and heterogeneity was also suggested by Cochran’s Q statistic and Egger intercept value |
|  | b) | Report any additional statistics (e.g., assessments of heterogeneity across genetic variants, such as *I^2^*, Q statistic or E-value) | 12  Table S7 | All directions of IVW results in MVMR were consistent with those of MVMR Egger sensitivity analyses results. Furthermore, a low probability of horizontal pleiotropy and heterogeneity was also suggested by Cochran’s Q statistic and Egger intercept value |
| 13 | **Sensitivity analyses and additional analyses** |  |  |  |
|  | a) | Report any sensitivity analyses to assess the robustness of the main results to violations of the assumptions | Table S7 |  |
|  | b) | Report results from other sensitivity analyses or additional analyses | Table S7 |  |
|  | c) | Report any assessment of direction of causal relationship (e.g., bidirectional MR) | 11 | On the contrary, there was barely any evidence showing the positive genetic prediction of AF on periodontal disease in both discovery and validation analysis (Table S4). |
|  | d) | When relevant, report and compare with estimates from non-MR analyses | 18 | A cohort from Japan histologically revealed that periodontal inflammation surface area was positively correlated with atrial fibrosis (R = 0.46, p < 0.0001) and remained significant (β = 0.016, p = 0.0002) after multivariable adjustment[55]. Another dental cohort reported similar results that severe periodontitis was associated with AF (adjusted hazard ratio 1.31; [95%CI, 1.06-1.62]), correspondingly, compared with episodically dental care users, regular users had a lower risk for AF (adjusted hazard ratio 0.88, [95% CI, 0.78-0.99]) |
|  | e) | Consider additional plots to visualize results (e.g., leave-one-out analyses) | Figure S1,2 |  |
|  | **DISCUSSION** |  |  |  |
| 14 | **Key results** | Summarize key results with reference to study objectives | 17-18 | This MR study assessed the association between periodontal disease and the higher risk of atrial fibrillation with OR of 1.16 considering genetic liability, and the positive causality remained significant after adjustment for dental caries and pulp diseases. Subsequently, the mediation analysis was conducted on the 25 factors, of which we ultimately determined 5 potential mediators and quantified their mediated proportions, including weight (30.3%), IL-17 (17.2%), TNF (14.08%), coronary atherosclerosis (13.4%) and hypertension (11.6%). |
| 15 | **Limitations** | Discuss limitations of the study, taking into account the validity of the IV assumptions, other sources of potential bias, and imprecision. Discuss both direction and magnitude of any potential bias and any efforts to address them | 22 | Admittedly, this study also had some limitations. First, in our analysis, we assumed a linear relationship between periodontal disease and AF in both univariable and multivariable MR analyses; however, more research is needed to explore any potential non-linear associations by introducing regression analysis or other statistical models. Second, we screened mediators mainly based on clinical practice and epidemic support, while other significant mediating pathways might be omitted and need further investigation. Third, the selected bias is caused by GWAS extracted by European populations from developed countries where oral hygiene is highly valued. Therefore, investigating the applicability of our findings to other ethnic groups or developing countries is of necessity. Fourth, the heterogeneity of SNPs due to the large number of IVs may potentially impact the robustness of MR results. |
| 16 | **Interpretation** |  |  |  |
|  | a) | Meaning: Give a cautious overall interpretation of results in the context of their limitations and in comparison with other studies | 22 | Admittedly, this study also had some limitations. First, in our analysis, we assumed a linear relationship between periodontal disease and AF in both univariable and multivariable MR analyses; however, more research is needed to explore any potential non-linear associations by introducing regression analysis or other statistical models. Second, we screened mediators mainly based on clinical practice and epidemic support, while other significant mediating pathways might be omitted and need further investigation. Third, the selected bias is caused by GWAS extracted by European populations from developed countries where oral hygiene is highly valued. Therefore, investigating the applicability of our findings to other ethnic groups or developing countries is of necessity. Fourth, the heterogeneity of SNPs due to the large number of IVs may potentially impact the robustness of MR results. |
|  | b) | Mechanism: Discuss underlying biological mechanisms that could drive a potential causal relationship between the investigated exposure and the outcome, and whether the gene-environment equivalence assumption is reasonable. Use causal language carefully, clarifying that IV estimates may provide causal effects only under certain assumptions | 19 | The hypothesized mechanism for the association between periodontal disease and AF mainly focused on the systemic inflammation instigated by dental plaque and its locally released cytokines; the entry of low-level bacteremia into the bloodstream and invasion of the heart, accompanied by autoimmunity against molecular structures expressed in the heart triggered by the host’s immune response to specific oral pathogens[54,56]. On top of that, the overactivation of the autonomous nervous system responding to chronic inflammation has been believed to prompt electrical remodelling in AF |
|  | c) | Clinical relevance: Discuss whether the results have clinical or public policy relevance, and to what extent they inform effect sizes of possible interventions | 22 | It is important to note that maintaining oral hygiene and accepting standardized periodontal treatment could work as a prevention and intervention of AF for patients suffering from periodontal disease. |
| 17 | **Generalizability** | Discuss the generalizability of the study results (a) to other populations, (b) across other exposure periods/timings, and (c) across other levels of exposure | NONE |  |
|  | **OTHER INFORMATION** |  |  |  |
| 18 | **Funding** | Describe sources of funding and the role of funders in the present study and, if applicable, sources of funding for the databases and original study or studies on which the present study is based | 23 | Funding: This work was supported by the National Natural Science Foundation of China (No. 82205096), and the Scientific and Technological Innovation Project of the China Academy of Chinese Medical Sciences (No.CI2021A00918 and No. CI2021A03318). |
| 19 | **Data and data sharing** | Provide the data used to perform all analyses or report where and how the data can be accessed, and reference these sources in the article. Provide the statistical code needed to reproduce the results in the article, or report whether the code is publicly accessible and if so, where | 23 | Supplementary Materials: The following supporting information can be downloaded online. |
| 20 | **Conflicts of Interest** | All authors should declare all potential conflicts of interest | 25 | All authors declare that the research was conducted in the absence of any commercial or financial relationships that could be construed as potential competing interests. |

This checklist is copyrighted by the Equator Network under the Creative Commons Attribution 3.0 Unported (CC BY 3.0) license.

1. Skrivankova VW, Richmond RC, Woolf BAR, Yarmolinsky J, Davies NM, Swanson SA, et al. Strengthening the Reporting of Observational Studies in Epidemiology using Mendelian Randomization (STROBE-MR) Statement. JAMA. 2021;under review.

2. Skrivankova VW, Richmond RC, Woolf BAR, Davies NM, Swanson SA, VanderWeele TJ, et al. Strengthening the Reporting of Observational Studies in Epidemiology using Mendelian Randomisation (STROBE-MR): Explanation and Elaboration. BMJ. 2021;375:n2233.
