# Supplementary material for: The 2-Step Mendelian Randomisation Study Assesses Genetic Causality and Potential Mediators of Periodontal Disease and Atrial Fibrillation
Source: Int Dent J. 2025 Feb 22;75(3):2093–103. doi: 10.1016/j.identj.2024.12.029 (PMC12142772; doi:10.1016/j.identj.2024.12.029)
Supplement: Supplementary file 2 [file mmc2.docx]

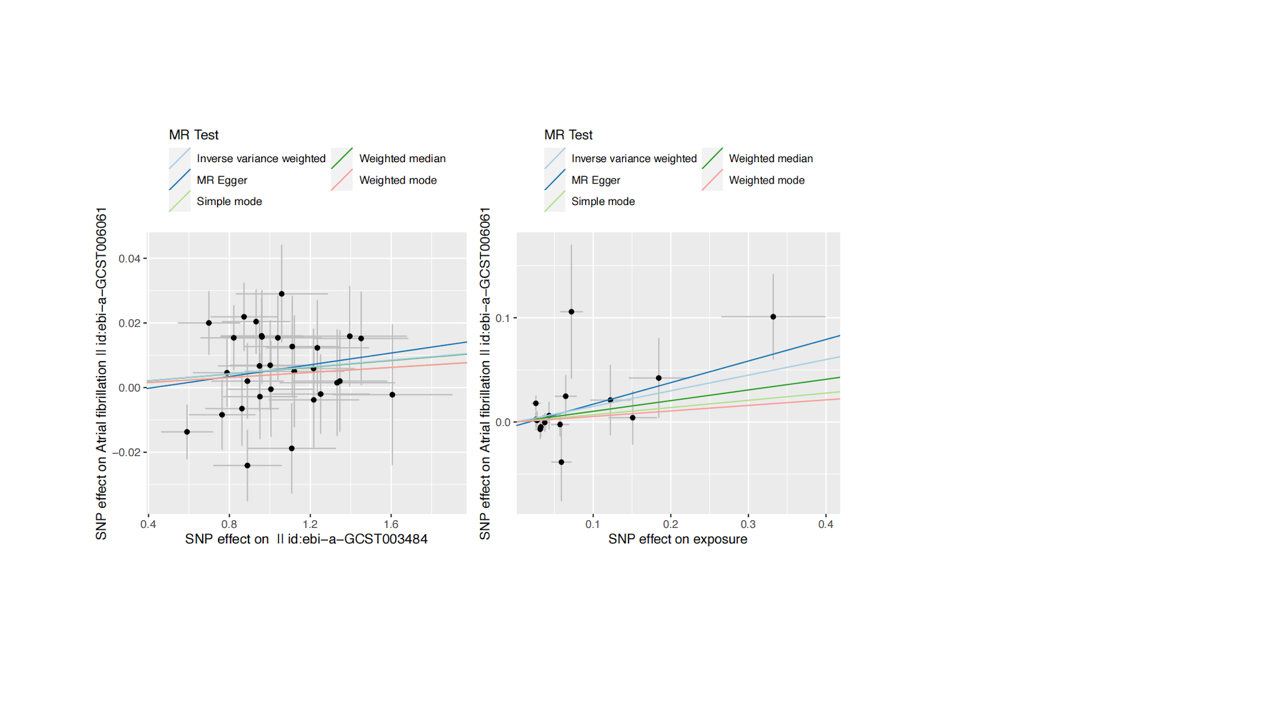


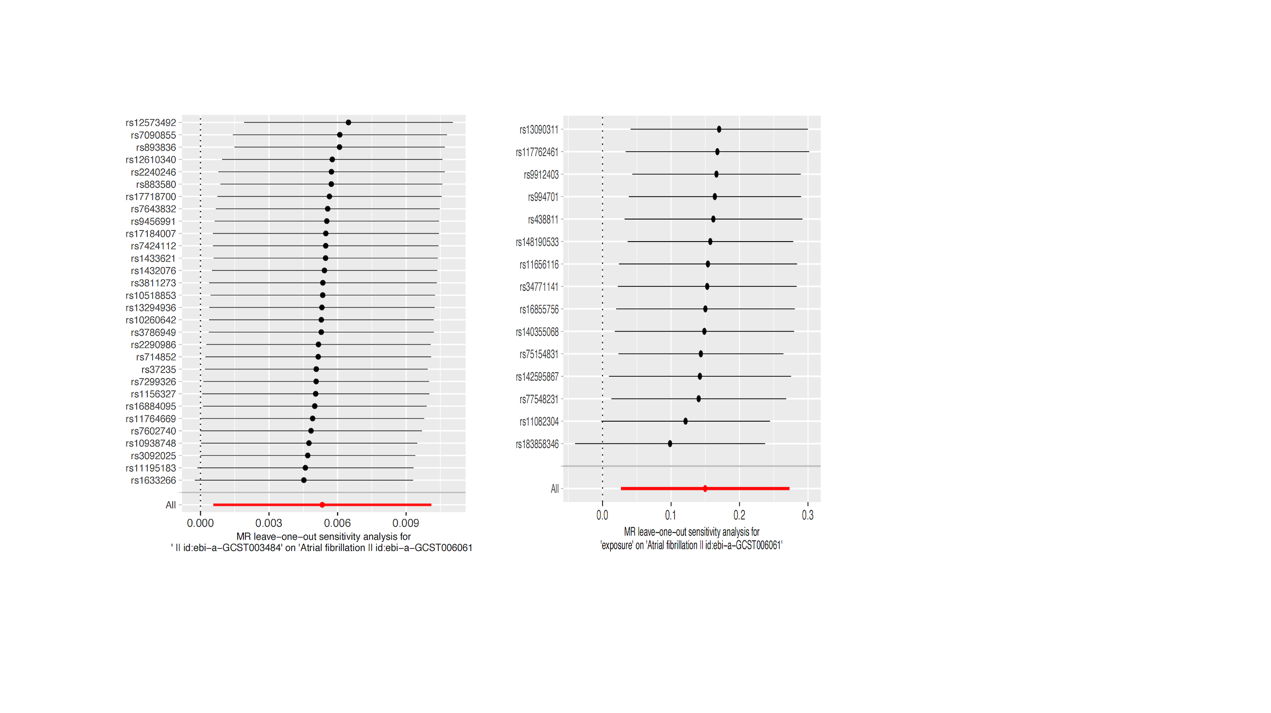


**Supplementary Figure Legends:**

Figure S1: The scatter diagram summarizing MR estimates using 5 methods of statistics. (A) The MR estimates of instrument variables extracted from the ARIC cohort of periodontal disease on the largest atrial fibrillation GWAS dataset; (B) The MR estimates of instrument variables extracted from the largest and the latest cohort of periodontal disease on the largest atrial fibrillation GWAS dataset. SNP, single nucleotide polymorphism

Figure S2: Leave-one-out plot for MR examining the outlier effect of PD on AF. (A) The discovery analysis of PD GWAS from the ARIC cohort; (B) The validation analysis of PD GWAS from the largest and the latest FinnGen. Results are expressed as an effect of AF per SD increase in PD. Abbreviations: AF, atrial fibrillation; GWAS, genome-wide association study, MR, Mendelian randomization; PD, periodontal disease
