# Supplementary material for: The 2-Step Mendelian Randomisation Study Assesses Genetic Causality and Potential Mediators of Periodontal Disease and Atrial Fibrillation
Source: Int Dent J. 2025 Feb 22;75(3):2093–103. doi: 10.1016/j.identj.2024.12.029 (PMC12142772; doi:10.1016/j.identj.2024.12.029)
Supplement: Supplementary file 3 [file mmc3.docx]

**Table S1 Evidenced-based support for the relationship between the 25 candidate mediators and atrial fibrillation**

| **Candidate Mediators** | **Epidemiological evidence** |
| --- | --- |
| **Inflammatory Biomarkers** | |
| CRP | A prospective study with follow-up for a mean of 6.9 +/- 1.6 (median 7.8) years indicated that baseline CRP (fourth versus first quartile) predicted a higher risk for developing future AF with an adjusted HR of 1.31(95% CI: 1.08, 1.58; P = 0.005). The HR of an elevated CRP for each 1-SD increase in developing future AF was 1.24 (95% CI: 1.11, 1.40; P < 0.001) ^[21]^. |
| WBC | A meta-analysis including 12 studies with 18,293 patients claimed that leucocyte telomere shortening was regarded as an independent predictor of recurrent AF (OR:4.32; 95% CI (2.42,7.69); P < 0.00001). Restoring LTL may provide a new treatment of AF in the future studies ^[22]^. |
| IL-1ß | Local inflammation acts an important role in persistent/ paroxysmal AF. The mRNA level of IL-1ß obtained from patients in CABG surgery was associated with persistent AF than the control group ((4.94 +/- 1.69 vs. 2.93 +/- 0.91, P < 0.01) ^[23]^. IL-6 can be used for assessing systematic inflammatory activity and correlates to mortality in AF, hazard ratios per 50% higher IL-6 at 2 months was 1.32 (95% CI:1.23, 1.41; P < .0001) in the ARISTOTLE trial ^[24]^. IL-17 is used to evaluate the probability of developing AF by regulating inflammation and fibrosis, and a study in China has proved that the correlation coefficient was 0.95(P<0.05) ^[25]^. |
| IL-6 |  |
| IL-17 |  |
| IL-18 | A prospective study (56 AF patients/26controls) showed that AF patient had higher levels of IL-18 than the control group ((471.50 +/- 144.91 vs. 232.20 +/- 55.33 pg/mL; P= 0.0001)). IL-18 was significantly association with AF (P < 0.0001, STB = 0.65) ^[26]^. |
| VEGF | VEGF from a whole cohort (59 AF and 40 without AF) was strongly related to the expression of adhesion molecules sCD40L (r=0.919; p<0.001), as a predictive marker for AF (p = 0.023) ^[27]^. |
| TNF | A clinical study included 67 patients that were scheduled to have heart surgery (31 AF group/36 controls). AF patients apparently had higher levels of TNF-α ((14.40±5.45) pg/ml vs. (4.20±3.19) pg/ml vs. (2.68±2.20) pg/ml, p=0.000)) than the controls ^[28]^. |
| **Weight** | A meta-analysis concluded that in the population-based cohort studies, obese individuals have an associated 49% increased risk of developing AF compared to nonobese individuals (RR 1.49, 95% CI 1.36-1.64); the overweight cohort had a 39% increased risk of developing AF (95% CI, 1.05-1.83) whereas the obese cohort had an 87% increased risk of developing AF (95% CI, 1.43-2.44) ^[29]^. |
| **Blood pressure** | |
| Hypertension | A meta-analysis of 31 studies including 9284 patients with essential hypertension confirmed the increased risk of AF when setting blood pressure level as matching criterion (4.00, 95% CI: 1.83-8.76) after median of 8.8 years ^[30]^. |
| SBP | In the LIFE study, the lower in-treatment SBP (per 10mmHg decrease) was associated with 17% risk reduction (P=0.008) for new-onset AF in ISH patients and 9% risk reduction (P=0.006) in non-ISH patients, a significant interaction (P=0.041) was in favor of SBP reduction and AF prevention in ISH vs. non-ISH patient ^[31]^. |
| DBP | In a cohort of initially healthy women, adjusted HR across baseline DBP categories (<65, 65 to 74, 75 to 84, 85 to 89, 90 to 94, and≥95 mmHg) were 1.0, 1.17 (95% CI, 0.81 to 1.69), 1.18 (95% CI, 0.84 to 1.65), 1.53 (95% CI, 1.05 to 2.23), 1.35 (95% CI, 0.82 to 2.22), and 2.15 (95% CI, 1.21 to 3.84) (P=0.004), which significantly correlates to the long term risk of AF ^[32]^. |
| **Metabolites** | |
| Blood glucose | A meta-analysis of diabetes/pre-diabetes and AF concluded that the summary RR was 1.20 (95% CI: 1.03-1.39, I-2 = 30%, n = 4, 2392 cases, 58,547 participants) for the association between prediabetes and atrial fibrillation; per 20 mg/dl increase of blood glucose in relation to atrial fibrillation (3385 cases, 247,447 participants) ^[33]^. |
| Fast Insulin | In a Chinese cohort, during the follow-up 1 year after ablation, 26.7% patients experienced AF recurrence, and the insulin resistance level estimated by the homeostasis model showed the independent association with AF recurrence (HR 1.259, 95% CI 1.086-1.460, P = 0.002) ^[34]^. |
| LDL-C | The hazard ratios of developing AF during the 35-year follow-up in LDL-C, HDL-C and Apolipoprotein A in 65,136 middle-aged individuals were 0.64 (95% CI: 0.45 to 0.92, p = 0.016), (1.13 (95% CI: 1.07 ~ 1.19, p < 0.001) to 1.53 (95% CI: 1.12 ~ 2.00, p = 0.007)), from the Swedish Apolipoprotein-Related Mortality Risk (AMORIS) cohort. High LDL-C in midlife patients was associated with a lower risk of AF, but low HDL-C and ApoA-I were associated with an increased risk of AF ^[35]^. |
| HDL-C |  |
| Apolipoprotein A |  |
| Coronary atherosclerosis | Rates of AF increase 40% to 50% in patients undergoing coronary bypass grafting. In an international analysis of 4624 participants without AF who underwent CABG with 5-year follow-up, and 778 (16.8%) patients had POAF that lasted for > 5 minutes; while increasing the use of proven secondary prevention medications was protective, being associated with a reduced risk of POAF. ^[36]^ |
| Vitamin C | A meta-analysis evaluating the benefits and adverse effects of oral vitamin C supplement on AF after cardiac surgery concludes that vitamin C treatment was associated with a substantial reduction in postoperative AF with OR 0.47(95% CI, 0.36-0.62; evidence rank: moderate) ^[37]^. |
| Vitamin D | A cohort study (102 NVAF patients/100 controls) suggested that 25-OH vitamin D level was an independent predictor of AF with HR of 0.86 (95% CI: 0.786-0.940, P= 0.001) ^[38]^. Whereas a study including 25119 participants without AF over a median of 5 years reported insufficient support of vitamin D in preventing AF with HR of 1.09 (95% CI: 0.96-1.25; P=0.19) ^[39]^. |
| **Coagulation function** | |
| PLT | This clinical study enrolled 19 consecutive patients with paroxysmal AF to receive remote IPC (n=10) or a sham intermittent ischemia (n=9) before receiving the RF catheter ablation. Remote IPC patients had a lower increase in MPA formation (p<0.0001), CD41 in the MPA gate (p=0.002) and CD41 (p<0.0001) and CD62 (p=0.002) in the platelet gate compared controls. The study therefore introduced the remote IPC can constantly reduce all markers of platelet ^[40]^. |
| PG | In a prospective study including 1,002 anticoagulated AF patients, followed for a median time of 25.7 months, the addition of tertiles of PGαto CHA2DS2-VASc score improved ROC curves for each outcome and cardiovascular events with net reclassification index of 0.24 (95%CI: 0.06–0.53, p=0.0067)^[41]^. |
| **Autonomic nerve function** | |
| Heart rate variability | Within a 3-year follow-up, 614 patients with permanent AF were randomly treated by lenient rate-control ( resting heart rate <110bpm) and strict rate-control (resting heart rate <80bpm), and the former therapy presents the non-inferiority with the latter; the incident of MACE was 12.9% in lenient-control and 14.9% in strict-control with an absolute difference of −2.0 % (90% CI: −7.6 to 3.5) and a hazard ratio of 0.84 (90% CI, 0.58 to 1.21, P=0.001) ^[42]^. |
| Major depression | A study investigating 101 AF patients based on the Beck Depression Inventory, at baseline, symptoms of depression prevailed in 38%, depression (r= 0.67, P < 0.001) was significantly correlated with quality of life at 6 months in the patients with AF, depression emerged as a predictor of AF patients’ quality of life (ß= 0.60, t =4.94, P < 0.001) ^[43]^. |
| Sleeplessness | In a meta-analysis of 10 studies, those who had insomnia carried a 1.3-fold increased risk of incident AF compared to individuals without sleeplessness. The odds ratio for the association between AF and Sleeplessness was 1.30 (95% CI, 1.26-1.35, I^2^=3%) ^[44]^. |

Abbreviations:

AF, atrial fibrillation; Ang, angiotensin; BNP, B-type natriuretic peptide; CABG, coronary artery bypass graft; CAMKII, calmodulin-dependent protein kinase; CI, confidence interval; CRP, C-reactive protein; DBP, diastolic blood pressure; PG, prostaglandin; HDL-C, high density lipoprotein cholesterol; HR, hazard ratio; IL, interleukin; IPC, ischemic preconditioning; ISH, isolated systolic hypertension; LDL-C, low density lipoprotein cholesterol; LTL, leucocyte telomere length; MACE, major adverse cardiovascular events; MPA, monocyte-platelet aggregate; NVAF, nonvalvular atrial fibrillation; OR, odds ratio; PLT, platelet; POAF, postoperative atrial fibrillation; RR, relative risk; SBP, systolic blood pressure; sCD40L, soluble CD40 ligand; SD, standard deviation; STB, total bilirubin; TNF, tumor necrosis factor; VEGF, vascular endothelial growth factor; WBC, white blood cell.

References:

21. Aviles RJ, Martin DO, Apperson-Hansen C, Houghtaling PL, Rautaharju P, Kronmal RA, et al. Inflammation as a Risk Factor for Atrial Fibrillation. Circulation. 2003,108,3006–10.

22. Zheng Y, Zhang N, Wang Y, Wang F, Li G, Tse G, et al. Association between leucocyte telomere length and the risk of atrial fibrillation: An updated systematic review and meta-analysis. Ageing Research Reviews. 2022,81,101707.

23.Liu Q, Zhang F, Yang M, Zhong J. Increasing Level of Interleukin-1β in Epicardial Adipose Tissue Is Associated with Persistent Atrial Fibrillation. Journal of Interferon & Cytokine Research. 2020,40,64–9.

24. Aulin J, Hijazi Z, Siegbahn A, Andersson U, Alexander JH, Connolly SJ, et al. Serial measurement of interleukin‐6 and risk of mortality in anticoagulated patients with atrial fibrillation: Insights from ARISTOTLE and RE‐LY trials. Journal of Thrombosis and Haemostasis. 2020;18(9):2287–95.

25. Fu XX, Zhao N, Dong Q, Du LL, Chen XJ, Wu QF, et al. Interleukin-17A contributes to the development of post-operative atrial fibrillation by regulating inflammation and fibrosis in rats with sterile pericarditis. International Journal of Molecular Medicine. 2015,36,83–92. doi: 10.3892/ijmm.2015.2204.s

26. Luan Y, Guo Y, Li S, Yu B, Zhu S, Li S, et al. Interleukin-18 among atrial fibrillation patients in the absence of structural heart disease. Europace. 2010,12,1713–8.

27. Choudhury A, Freestone B, Patel J, Lip GY. Relationship of soluble CD40 ligand to vascular endothelial growth factor, angiopoietins, and tissue factor in atrial fibrillation: a link among platelet activation, angiogenesis, and thrombosis?. Chest. 2007, 132, 1913-1919.

28. Deng H, Xue YM, Zhan XZ, Liao HT, Guo HM, Wu SL. Role of tumor necrosis factor-alpha in the pathogenesis of atrial fibrillation. Chin Med J (Engl). 2011,124, 1976-1982.

29. Wanahita N, Messerli FH, Bangalore S, Gami AS, Somers VK, Steinberg JS. Atrial fibrillation and obesity—results of a meta-analysis. American Heart Journal. 2008,155, 310–5.

30. Monticone S, D’Ascenzo F, Moretti C, Williams TA, Veglio F, Gaita F, et al. Cardiovascular events and target organ damage in primary aldosteronism compared with essential hypertension: a systematic review and meta-analysis. The Lancet Diabetes & Endocrinology. 2018,6,41–50.

31. Larstorp ACK, Stokke IM, Kjeldsen SE, Hecht Olsen M, Okin PM, Devereux RB, et al. Antihypertensive therapy prevents new-onset atrial fibrillation in patients with isolated systolic hypertension: the LIFE study. Blood Pressure. 2019,28, 317–26.

32. Conen D, Tedrow UB, Koplan BA, Glynn RJ, Buring JE, Albert CM.. Influence of Systolic and Diastolic Blood Pressure on the Risk of Incident Atrial Fibrillation in Women. Circulation. 2009,119, 2146–52.

33. Aune D, Feng T, Schlesinger S, Janszky I, Norat T, Riboli E. Diabetes mellitus, blood glucose and the risk of atrial fibrillation: A systematic review and meta-analysis of cohort studies. Journal of Diabetes and its Complications. 2018,32, 501–11.

34. Wang Z, Wang YJ, Liu ZY, Li Q, Kong YW, Chen YW, et al. Effect of Insulin Resistance on Recurrence after Radiofrequency Catheter Ablation in Patients with Atrial Fibrillation. Cardiovascular Drugs and Therapy. 2023, 37, 705–713. doi:10.1007/s10557-022-07317-z.

35. Ding M, Wennberg A, Gigante B, Walldius G, Hammar N, Modig K. Lipid levels in midlife and risk of atrial fibrillation over 3 decades-Experience from the Swedish AMORIS cohort: A cohort study. PLoS Med. 2022,19,e1004044.

36. Conen D, Wang MK, Devereaux PJ, Whitlock R, McIntyre WF, Healey JS, et al. (2021). New‐Onset Perioperative Atrial Fibrillation After Coronary Artery Bypass Grafting and Long‐Term Risk of Adverse Events: An Analysis From the CORONARY Trial. Journal of the American Heart Association, 2021, 10, e020426. doi:10.1161/JAHA.120.020426.

37. Hu X, Yuan L, Wang H, Li C, Cai J, Hu Y, et al. Efficacy and safety of vitamin C for atrial fibrillation after cardiac surgery: A meta-analysis with trial sequential analysis of randomized controlled trials. International Journal of Surgery. 2017, 37, 58–64.

38. Demir M, Uyan U, Melek M. The Effects of Vitamin D Deficiency on Atrial Fibrillation. Clin Appl Thromb Hemost.2014, 20, 98–103.

39. Albert CM, Cook NR, Pester J, Moorthy MV, Ridge C, Danik JS, et al. Effect of Marine Omega-3 Fatty Acid and Vitamin D Supplementation on Incident Atrial Fibrillation: A Randomized Clinical Trial. JAMA. 2021, 325,1061.

40. Stazi A, Scalone G, Laurito M, Milo M, Pelargonio G, Narducci M, et al. Effect of Remote Ischemic Preconditioning on Platelet Activation and Reactivity Induced by Ablation for Atrial Fibrillation. Circulation, 2014,129, 11–17

41. Pignatelli P, Pastori D, Carnevale R, Farcomeni A, Cangemi R, Nocella C, et al. Serum NOX2 and urinary isoprostanes predict vascular events in patients with atrial fibrillation. Thromb Haemost. 2015;113(03):617–24.

42. Van Gelder IC, Groenveld HF, Crijns HJGM, Tuininga YS, Tijssen JGP, Alings AM, et al. Lenient versus Strict Rate Control in Patients with Atrial Fibrillation. N Engl J Med. 2010, 362, 1363–73.

43. Chokesuwattanaskul R, Thongprayoon C, Sharma K, Congrete S, Tanawuttiwat T, Cheungpasitporn W. Associations of sleep quality with incident atrial fibrillation: a meta-analysis. Intern Med J. 2018, 48,964-972.

44. Thrall G, Lip GYH, Carroll D, Lane D. Depression, Anxiety, and Quality of Life in Patients With Atrial Fibrillation. Chest. 2007, 32, 1259–64.

45. Zhang J, Chen Z, Pärna K, Van Zon SKR, Snieder H, Thio CHL. Mediators of the association between educational attainment and type 2 diabetes mellitus: a two-step multivariable Mendelian randomisation study. Diabetologia. 2022,65, 1364–74.
